# Supplementary material for: Hyperthermic Intraperitoneal Chemotherapy (HIPEC): New Approaches and Controversies on the Treatment of Advanced Epithelial Ovarian Cancer—Systematic Review and Meta-Analysis
Source: J Clin Med. 2023 Nov 9;12(22):7012. doi: 10.3390/jcm12227012 (PMC10672052; doi:10.3390/jcm12227012)
Supplement: Supplementary file 1 [file jcm-12-07012-s001.zip › jcm-2690031-supplementary.pdf]

Table S1. Quality scores of the case-control studies included in the meta-analysis, assessed by the Newcastle-Ottawa scale.

Newcastle-Ottawa scale for assessment of quality of included studies - Case-control studies (each asterisk represents if individual criterion within the subsection was fulfilled).

		Selection				Comparability		Exposure			
**Author**	**Year**	**Case definition**	**Repres-en-tativen-ess of the cases**	**Selecti-on of Controls**	**Definit-ion of Controls**	**Control for the most import-ant factor**	**Control for any additi-onal factors**	**Ascert-ainme-nt of exposu-re**	**Same method for cases and controls**	**Non-Respo-nse Rate**	**Overall quality**
Ceresoli [29]	2018	*	*	0	0	*	*	*	*	*	**7**
He [25]	2021	*	*	*	*	*	*	*	*	0	**8**
Lei [34]	2020	*	*	0	*	*	*	*	*	0	**7**
Lim [27]	2022	*	*	*	*	*	*	*	*	0	**8**
Marrelli [31]	2021	*	*	0	0	*	*	*	*	*	**7**
Mendivil [18]	2017	*	*	*	*	*	*	*	*	0	**8**
Van Driel [12]	2018	*	*	*	*	*	*	*	*	0	**8**
Wu [30]	2022	*	*	*	*	*	*	*	*	0	**8**
Ghirardi [22]	2022	*	*	*	0	*	*	*	*	0	**7**
Cascales Campos [20]	2014	*	*	0	0	*	*	*	*	*	**7**
Cascales Campos [21]	2015	*	*	0	0	*	*	*	*	*	**7**
Zivanovic [19]	2021	*	*	*	*	*	*	*	*	*	**9**
Safra [23]	2014	*	*	0	*	*	*	*	*	0	**7**
Fagotti [26]	2012	*	*	*	*	*	*	*	*	0	**8**
Baiocchi [33]	2015	*	*	*	*	*	*	0	*	*	**8**
Munoz-Casares [32]	2009	*	*	*	0	*	*	0	*	*	**6**
Biacchi [24]	2019	*	*	0	*	*	*	*	*	*	**8**

Table S2: Quality Assessment for RCTs* (Method: Cochrane Collaboration's Tool for Assessing Risk of Bias)

	Selection bias		Performance bias	Detection bias	Attrition bias	Reporting bias	Other bias	Total
**Author**	**Random sequence generation**	**Allocation concealment**	**Binding of participant and personnel**	**Blinding of outcome assessment**	**Incomplete outcome data**	**Selective reporting**	**Anything else, ideally prespecifed**	**Low on risk of bias**
Spiliotis [14]	Low	Low	Low	Low	Low	Unclear	Low	7-Jun

RCTs*=randomized controlled trials
